# Promoting Science Literacy by Engaging the Public

**DOI:** 10.1371/journal.pbio.0030427

**Published:** 2005-12-13

**Authors:** Anna Liem

## Abstract

The Science Adventure Center at Hawaii's Bishop Museum draws visitors into the excitement of scientific discovery and knowledge.

When you're standing inside a volcano, it's hard not to pay attention to what's around you. And whose interest wouldn't be captured by guiding remotely operated vehicles through a hydrothermal vent or pumping magma into a volcano and watching it erupt? The new Science Adventure Center at the Bishop Museum in Honolulu, Hawai'i, captures the imagination in a bid to increase science literacy. In doing so, the center provides a valuable opportunity not just for its visitors, but for all those interested in science education. Both the center's mission and its design inspire critical questions: What is science literacy? How does a person become science-literate? What roles can museums, schools, and other institutions play in promoting science literacy? And what can teachers learn from museums, and vice versa, about science education?

## What Is Science Literacy?

Science literacy is much more than the memorization or even comprehension of scientific facts and principles. The American Association for the Advancement of Science defines the science-literate person as “one who is aware that science, mathematics, and technology are interdependent human enterprises with strengths and limitations; understands key concepts and principles of science; is familiar with the natural world and recognizes both its diversity and unity; and uses scientific knowledge and scientific ways of thinking for individual and social purposes” [1]. Thus defined, science literacy encompasses not only the knowledge and understanding of scientific ideas and processes, but also, crucially, the ability and desire to apply those ideas and processes. Increasing the public's science literacy requires much more than the transmission of information; it must also change people's attitudes and actions.

**Figure:**
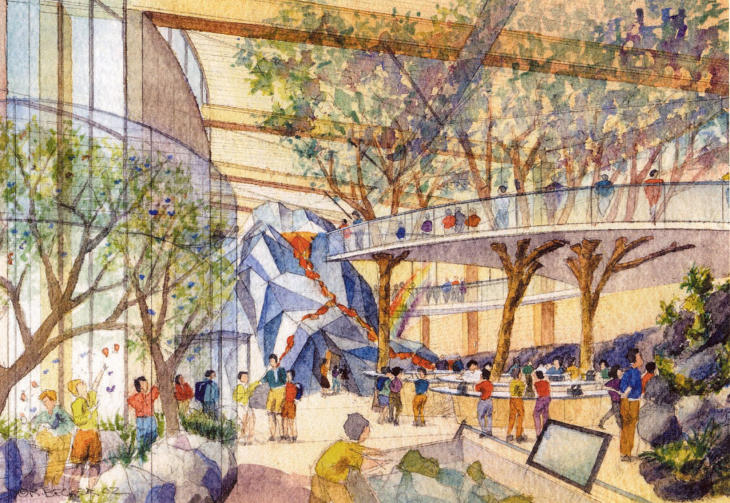
The Science Adventure Center at the Bishop Museum in Honolulu, Hawai'i (Image: Bishop Museum)

## Promoting Science Literacy: Engagement

To effect change, one must first attract interest. At the Science Adventure Center, large-scale multi-sensory displays do just that: they grab attention. At the Hot Spot Theater, a furnace used to melt lava rock creates a literal hot spot that visitors can feel from the other end of the room, while ceiling panels with a rippled texture and moving red lights create the impression of standing inside a volcano. The more senses you stimulate, the more likely you are to engage your audience; science teachers use this same philosophy when designing lessons with visual, auditory, and/or tactile elements. And when resources permit, primary school classrooms are usually enriched with posters, three-dimensional displays, and student work, a strategy that has also been adopted by secondary school teachers and is slowly filtering into college classrooms.

At museums, visitors also gravitate towards interactive exhibits like the center's wave-making tank, where museum-goers can trigger wind-generated surf, earthquake-generated tsunamis, and landslide-generated mega-tsunamis. In the classroom, students are likewise more apt to be engaged by interactive lessons—hence the popularity of hands-on activities. In the most complete form of student-directed learning, students ask and investigate their own questions, perhaps by searching for information on the Internet or by designing and executing their own experiments.

The Science Adventure Center also engages people's interest by tapping into their prior knowledge. A familiar context not only helps to engage one's attention, it also creates an experience more likely to produce lasting change. Constructivist learning theory suggests that new knowledge and understanding is built upon prior knowledge. Since it's located in Hawai'i, the center focuses on the islands' unique natural environment and draws on residents' experience of news stories about Kilauea volcano, the daily surf report, and even the rainbow decorating the state's license plates. Teachers do the same thing by selecting topics that are relevant to their students. Even within the framework of the National Science Education Standards, teachers can meet national standards with topics of local interest. For example, teachers in Hawai'i might address the standard of “biological evolution” with a study of the Hawaiian honeycreepers, an outstanding example of adaptive radiation.

The activation of prior knowledge is a critical part of any educational experience, whether that experience seeks to replace, alter, or augment an individual's existing understanding [2]. For example, the center confronts people's stereotyped images of volcanos with side-by-side representations of a shield volcano (Mauna Loa) and a composite volcano (Mount Ngauruhoe, which visitors may recognize as Mount Doom from Peter Jackson's movie adaptations of *The Lord of the Rings*). The display simply wouldn't work if it didn't trigger visitors' prior images of volcanoes.

## Partnerships between Museums and Schools

Engaging the interest of students and the public is a necessary component to promoting science literacy, but it is not sufficient by itself. Museum exhibits, while often extremely attention-grabbing, are also usually limited in duration; visitors, particularly school groups, frequently visit an exhibit only once or twice a year. A highly engaging exhibit might draw people back again and again or inspire visitors to learn more after they leave, but museums can also partner with schools to create more extensive learning experiences.

The designers of the Science Adventure Center deliberately chose topics that align with the state's science content standards, and geared many of their displays towards the associated grade level. They solicited input from teachers in Hawai'i during the design process, and teachers and students were also involved in the actual construction of the Origins Tunnel, an exploration of Native Hawaiian creation myths. In this black-lit corridor, the walls are decorated with student-created artwork that illustrates the Hawaiian chants vibrating through the tunnel. The Bishop Museum also frequently produces specific curriculum materials for use with their exhibits, and often involves teachers in the generation of those materials. Close partnerships between museums and schools mean that students can become engaged by visiting an exhibit and then deepen their knowledge and understanding in the classroom—the ideal combination.
